# Comparison of natural orifice specimen extraction surgery and conventional laparoscopic-assisted resection in the treatment effects of low rectal cancer

**DOI:** 10.1038/s41598-021-88790-8

**Published:** 2021-04-29

**Authors:** Yihao Zhu, Huan Xiong, Yinggang Chen, Zheng Liu, Zheng Jiang, Rui Huang, Feng Gao, Qian Zhang, Meng Wang, Yinghu Jin, Tianyu Qiao, Tianyi Ma, Hanqing Hu, Xishan Wang, Qingchao Tang, Guiyu Wang

**Affiliations:** 1 Department of Colorectal Surgery, The Second Affiliated Hospital of Harbin Medical University, Harbin, 150081 China; 2 Department of Colorectal Surgery, National Cancer Center, National Clinical Research Center for Cancer, Cancer Hospital, Chinese Academy of Medical Sciences and Peking Union Medical College, Beijing, 100021 China; 3 Department of Colorectal Surgery, Zhejiang Cancer Hospital (Affiliated Cancer Hospital of the Chinese Academy of Sciences), Hangzhou, 310022 China

**Keywords:** Cancer, Cancer

## Abstract

Natural orifice specimen extraction surgery (NOSES) is an intra-abdominal procedure that does not require an auxiliary incision to take a surgical sample from the abdominal wall through the natural orifice, but there are few systematic clinical studies on it. The aim of this study was to demonstrate the safety and feasibility of NOSES. We retrospectively analyzed the clinical data and follow-up of 165 patients with low rectal cancer who underwent NOSES or conventional laparoscopic surgery at our center from January 2013 to June 2015. From the perioperative data and postoperative follow-up results of both groups, patients in the NOSES group had less intraoperative bleeding (49.3 ± 55.8 ml vs*.* 75.1 ± 57.3 ml, *p* = 0.02), shorter postoperative gastrointestinal recovery (42.3 ± 15.5 h vs*.* 50.1 ± 17.0 h, *p* = 0.01), less postoperative analgesic use (35.6% vs*.* 57.6%, *p* = 0.02), lower postoperative pain scores, lower rate of postoperative complications (6.8% vs*.* 25.4%, *p* = 0.01), better satisfaction of the image and cosmesis of the abdominal wall postoperatively, and higher quality of life. Moreover, there was no significant difference in overall survival (OS) and disease-free survival (DFS) between two groups. Overall, NOSES is a safe and reliable minimally invasive surgical technique for patients with low rectal cancer.

## Introduction

Colorectal cancer (CRC) is the third most common malignancy in the world and the fourth most common cause of cancer-related deaths^1^. In the past few decades, approximately 1 million new cases of colorectal cancer have been diagnosed in the worldwide^2^. In the past ten years, due to the progress in diagnosis and management of CRC, the mortality rate of CRC has been reduced by more than 20%^3^. Unfortunately, colorectal cancer still causes more than 600,000 deaths per year^4^. At present, the main treatment methods of colorectal cancer are surgery, adjuvant chemotherapy and radiotherapy^5^. And the choice of surgical methods, also from the initial open surgery, to the current mature laparoscopic technology^6^. However, conventional laparoscopic surgery requires an auxiliary incision in the abdominal wall to complete the extraction of specimens, which will also cause postoperative pain, increase the risk of incision complications, affect the aesthetic effect of the abdominal wall, and even bring long-term adverse psychological implications to patients. How to avoid the auxiliary incision of conventional laparoscopic surgery has been the main obstacle to be overcome in conventional laparoscopic surgery. Therefore, NOSES comes out. NOSES is an intra-abdominal procedure performed using conventional laparoscopic instruments, transanal endoscopic microsurgery (TEM) or soft endoscopy, and the sample is taken from the abdominal wall through the natural cavity (rectum or vagina) without an auxiliary incision^7^. Recently, NOSES has become a new minimally invasive surgical modality. NOSES is preformed totally under laparoscopy and the specimen is extracted from natural orifice (oral, vaginal or anus) which can avoid wound-related complications and perform recovery more rapidly. The technique has been successfully used in the treatment of gastric, colon and rectal cancer.

However, the development of NOSES is in the phase of exploration to see whether this technique is feasible and safe, and strong evidence-based evidence is lacking at this stage. This study further demonstrated the safety and feasibility of NOSES in the treatment of colorectal cancer by retrospectively analyzing the short-term and long-term effects of the low-rectal cancer surgery performed by our center, and provided more real and objective evidence-based medical evidence for the promotion and development of NOSES.

## Results

### Propensity-score matching results of the two groups of patients

In this study, we performed a propensity score matching (PSM) on the gender, age, body mass index (BMI), American Society of Anesthesiologists score (ASA score), preoperative carcinoembryonic antigen (CEA), T stage, N stage, and preoperative pelvic floor distress inventory-short form 20 (PFDI-20) scores for the two groups (Table 1). The two groups of patients were basically consistent in these basic data.

**Table 1:** Baseline information for two groups of patients.

Characteristics	Before PSM			After PSM		
	CLR (N = 106)	NOSES (N = 59)	*p* value	CLR (N = 59)	NOSES (N = 59)	*p* value
Gender (N,%)			0.16			0.46
Male	64(60.4%)	29(49.2%)		33(44.1%)	29(49.2%)	
Female	42(39.6%)	30(50.8%)		26(55.9%)	30(50.8%)	
Age (years)^a^	59.8 ± 10.4	59.6 ± 12.1	0.92	60.4 ± 9.7	59.6 ± 12.1	0.69
BMI (kg/m^2^)^a^	23.0 ± 2.9	23.3 ± 2.7	0.40	23.1 ± 2.9	23.3 ± 2.7	0.66
ASA grade (N,%)			0.76			0.81
I/II	86(81.1%)	49(83.1%)		48(81.4%)	49(83.1%)	
III	20(18.9%)	10(16.9%)		11(18.6%)	10(16.9%)	
Preoperative CEA (N,%)^b^			0.76			0.80
Positive	20(18.9%)	10(16.9%)		9(15.3%)	10(16.9%)	
Negative	86(81.1%)	49(83.1%)		50(84.7%)	49(83.1%)	
T Stage (N,%)			0.27			0.71
Tis/T1	20(18.9%)	14(23.7%)		11(18.6%)	14(23.7%)	
T2	29(27.3%)	21(35.6%)		20(33.9%)	21(35.6%)	
T3	57(53.8%)	24(40.7%)		28(47.5%)	24(40.7%)	
N Stage (N,%)			0.76			0.68
N0	73(68.9%)	42(71.2%)		44(74.6%)	42(71.2%)	
N1/N2	33(31.1%)	17(28.8%)		15(25.4%)	17(28.8%)	
Preoperative PFDI-20^a^	7.11 ± 2.11	6.98 ± 1.65	0.68	7.05 ± 2.01	6.98 ± 1.65	0.84

^a^Mean ± SD.

^b^The cut-off value was considered to be 5 ng/ml.

### Comparison of short-term efficacy between NOSES group and CLR group

**Figure 1:**
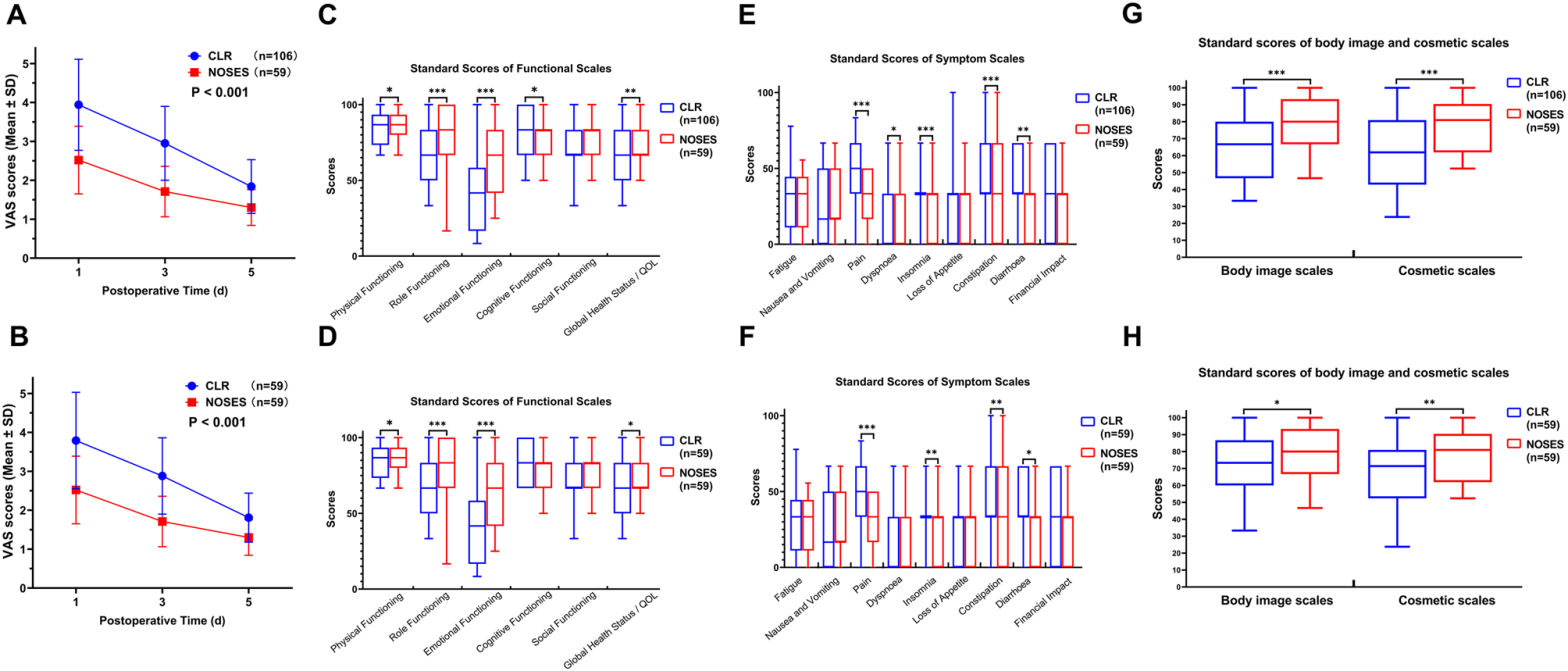
Comparison of short-term curative effect between two groups of patients. (**A**, **B**) Postoperative analgesic drug use in two groups of patients. (**A**) Before PSM. (**B**) After PSM. (**C**–**F**) EORCT Quality of Life questionnaire-Core 30 results of two groups. (**C**) Functional Scales (before PSM). (**D**)Symptom Scales (before PSM). (**E**) Functional Scales (after PSM). (**F**) Symptom Scales (after PSM). (**G**, **H**) Scores of body image and cosmetic scales (Higher scores indicate better body image and higher satisfaction with scars). (**G**) Before PSM. (**H**) After PSM. (*p < 0.05, **p < 0.01, ***p < 0.001).

We investigated the short-term efficacy of NOSES by reviewing our database and follow-up. First, we can see that the amount of blood loss in the NOSES group was significantly less than that in the CLR group. It is worth noting that the NOSES group was superior to the CLR group in terms of postoperative gastrointestinal function recovery time. Meanwhile, patients in the NOSES group used significantly fewer additional analgesics postoperatively and had significantly lower pain scores compared to patients in the CLR group (Fig. 1A,B). Looking at the postoperative complications, we can see that the complications associated with incision in the NOSES group are significantly less than the CLR group (Table 2). Moreover, from the six-month postoperative anal function and PFDI-20 scores, there was no significant difference in anal function between the NOSES group and the CLR group (Table 3). And the postoperative symptoms of lower urinary tract, lower gastrointestinal tract, and pelvic organ prolapse in the NOSES group were consistent with those before surgery (Table 4). However, in terms of quality of life, the NOSE group was significantly better than the CLR group in functional aspects such as physical functioning, role functioning, emotional functioning, global health status and in symptoms such as pain, insomnia constipation, and diarrhoea (Fig. 1C–F). What's more, in terms of the satisfaction of the appearance of the abdominal wall, the NOSES group was significantly better than the CLR group (Fig. 1G,H).

**Table 2:** Comparison of postoperative conditions between the two groups.

Outcome	Before PSM			After PSM		
	CLR (N = 106)	NOSES (N = 59)	*p* value	CLR (N = 59)	NOSES (N = 59)	*p* value
Operative time (min)^a^	193 ± 55	190 ± 43	0.71	194 ± 54	190 ± 43	0.66
Blood loss (mL)^a^	87.7 ± 98.6	49.3 ± 55.8	0.007	75.1 ± 57.3	49.3 ± 55.8	0.02
Length of abdominal incision (cm)^a^	8.7 ± 1.2	1.3 ± 0.3	< 0.001	5.8 ± 0.8	1.3 ± 0.3	< 0.001
Number of dissected lymph nodes (pieces)^a^	13.4 ± 4.7	12.7 ± 4.4	0.36	13.5 ± 4.8	12.7 ± 4.4	0.31
Positive Lymph node (pieces)^a^	1.2 ± 2.6	0.6 ± 1.3	0.15	0.9 ± 2.2	0.6 ± 1.3	0.53
Positive margin (N,%)	0(0)	0(0)	NA	0(0)	0(0)	NA
Intraoperative complications (N,%)	0(0)	0(0)	NA	0(0)	0(0)	NA
Grade (N,%)			0.98			0.90
Well-differentiated	22(20.8%)	12(20.3%)		11(18.6%)	12(20.3%)	
Moderately-differentiated	76(71.7%)	42(71.2%)		44(74.6%)	42(71.2%)	
Poor-differentiated	8(7.5%)	5(8.5%)		4(6.8%)	5(8.5%)	
Histology (N,%)			0.06			0.11
Adenocarcinoma	100(94.3%)	49(83.1%)		56(94.9%)	49(83.1%)	
Tubular adenocarcinoma	1(0.9%)	2(3.4%)		1(1.7%)	2(3.4%)	
Mucinous	5(4.8%)	8(13.5%)		2(3.4%)	8(13.5%)	
Usage of additional analgesics (N,%)	57(53.8%)	21(35.6%)	0.03	34(57.6%)	21(35.6%)	0.02
VAS score^a^			< 0.001^b^			< 0.001^b^
Day 1 postoperatively	3.94 ± 1.17	2.52 ± 0.87		3.79 ± 1.24	2.52 ± 0.87	
Day 3 postoperatively	2.95 ± 0.95	1.71 ± 0.65		2.88 ± 0.98	1.71 ± 0.65	
Day 5 postoperatively	1.84 ± 0.69	1.30 ± 0.46		1.81 ± 0.63	1.30 ± 0.46	
Gastrointestinal function recovery time (hour)^a^	53.5 ± 25.8	42.3 ± 15.5	< 0.001	50.1 ± 17.0	42.3 ± 15.5	0.01
Postoperative hospital stay (day)^a^	13.8 ± 5.2	10.9 ± 2.4	< 0.001	14.5 ± 5.5	10.9 ± 2.4	< 0.001
Postoperative complication (N,%)	21(19.8%)	4(6.8%)	0.03	15(25.4%)	4(6.8%)	0.006
Anastomotic leakage (N,%)	4(3.8%)	3(5.1%)	0.70	4(6.8%)	3(5.1%)	1.00
Intra-abdominal infection (N,%)	2(1.9%)	0(0)	0.54	2(3.4%)	0(0)	0.48
Ileus (N,%)	2(1.9%)	0(0)	0.54	1(1.7%)	0(0)	1.00
Pneumonia (N,%)	1(0.9%)	1(1.7%)	1.00	1(1.7%)	1(1.7%)	1.00
Pulmonary embolism (N,%)	0(0)	0(0)	NA	0(0)	0(0)	NA
Incision-related complications (N,%)	12(11.3%)	0(0)	0.01	7(11.9%)	0(0)	0.01
Bleeding (N,%)	2(1.9%)	0(0)	/	2(3.4%)	0(0)	/
Fever (N,%)	3(2.8%)	0(0)	/	1(1.7%)	0(0)	/
Infection (N,%)	2(1.9%)	0(0)	/	1(1.7%)	0(0)	/
Fat liquefaction (N,%)	4(3.8%)	0(0)	/	3(5.1%)	0(0)	/
Incisional hernia (N,%)	1(0.9%)	0(0)	/	0(0)	0(0)	/
Postoperative PFDI-20 score^a^	6.51 ± 1.83	6.56 ± 1.71	0.86	6.43 ± 1.79	6.56 ± 1.71	0.69
Reoperation (N,%)	2(1.9%)	1(1.7%)	1.00	2(3.4%)	1(1.7%)	1.00
Medical expenses (RMB)^a^	69,614 ± 17,535	69,258 ± 19,381	0.91	70,834 ± 20,656	69,258 ± 19,381	0.67

^a^Mean ± SD.

^b^The *p* value was calculated by a two-way repeated measures ANOVA.

**Table 3:** Comparison of PFDI-20 before and after surgery in NOSES group of patients.

	Preoperative	Postoperative	*p* value
POPDI-6	2.02 ± 0.80	1.86 ± 0.94	0.34
CRADI-8	3.42 ± 1.19	3.14 ± 1.21	0.29
UDI-6	1.54 ± 0.93	1.56 ± 0.95	0.92
PFDI-20	6.98 ± 1.65	6.56 ± 1.71	0.17

PFDI-20 is made up of those three parts: Pelvic organ prolapse distress inventory 6 (POPDI-6); Colorectal, anal distress inventory 8 (CRADI,8); Urinary distress inventory 6 (UDI-6).

**Table 4:** Postoperative Wexner scores in both groups.

Type of incontinence	Before PSM			After PSM		
	LA (N = 106)	NOSES (N = 59)	*p* value	LA (N = 59)	NOSES (N = 59)	*p* value
Solid^a^	2.32	2.41	0.46	2.08	2.41	0.11
Liquid^a^	2.08	2.29	0.23	2.19	2.29	0.42
Gas^a^	3.39	3.31	0.60	3.00	3.31	0.10
Wears pad^a^	0.05	0.08	0.43	0.07	0.08	0.73
Lifestyle alteration^a^	2.23	2.08	0.12	2.12	2.08	0.65

^a^Mean.

### Long-term efficacy comparison between NOSES group and CLR group

**Figure 2:**
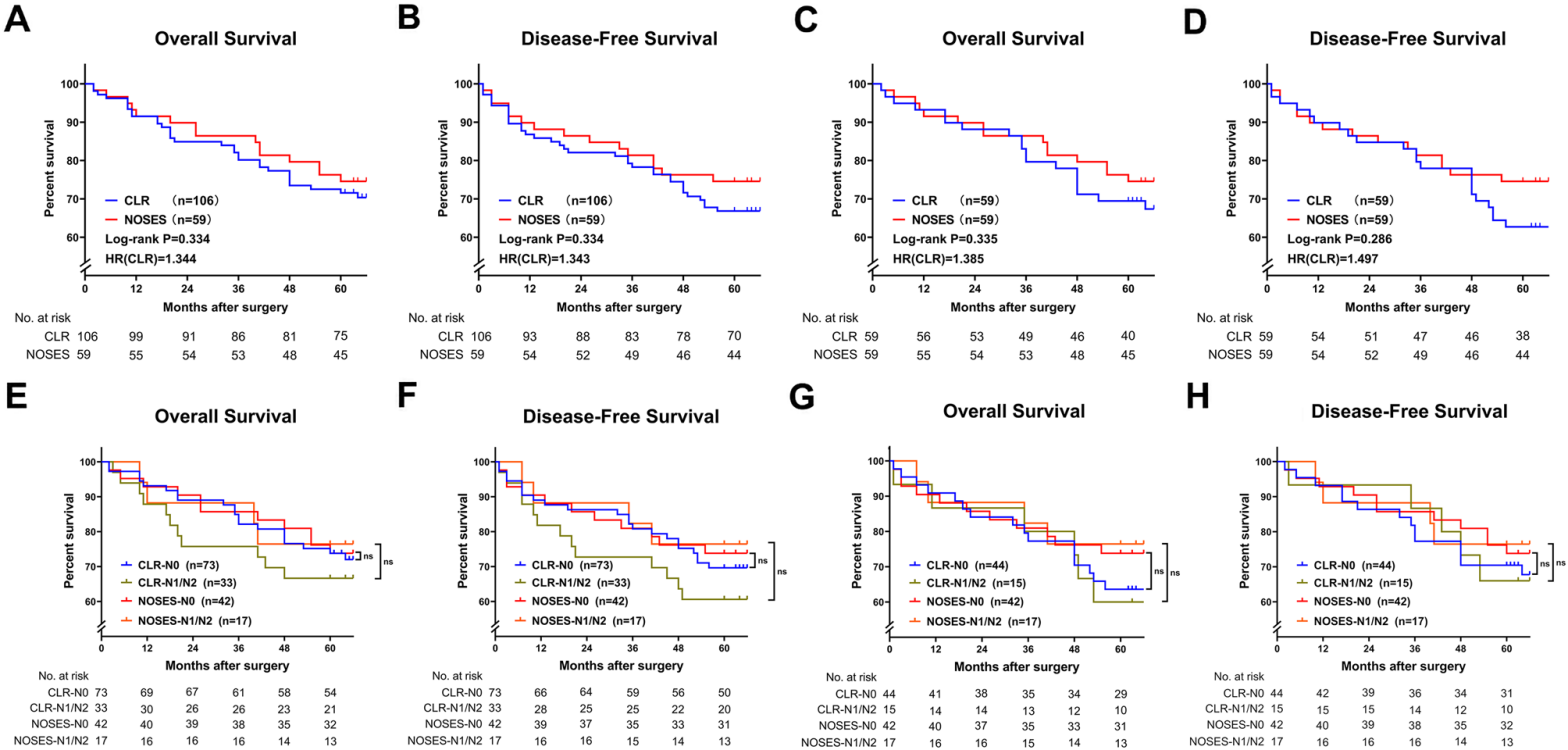
Comparison of long-term curative effect between two groups of patients. (**A**–**D**) Overall survival and Disease-Free survival between two groups. (**A**, **B**) Before PSM. (**C**, **D**) After PSM. (**E**–**G**) Comparison of overall survival and disease-free survival of patients with different N stages in the two groups. (**E**) overall survival before PSM: Log-rank p (CLR-N0 vs. NOSES-N0) = 0.853, HR(CLR-N0/NOSES-N0) = 1.071, Log-rank p (CLR-N1/N2 vs. NOSES-N1/N2) = 0.151, HR(CLR-N1/N2/NOSES-N1/N2) = 2.164. (**F**) disease-free survival before PSM: Log-rank p (CLR-N0 vs. NOSES-N0) = 0.694, HR(CLR-N0/NOSES-N0) = 1.155, Log-rank p (CLR-N1/N2 vs. NOSES-N1/N2) = 0.276, HR(CLR-N1/N2/NOSES-N1/N2) = 1.839. (**G**) Overall survival after PSM: Log-rank p (CLR-N0 vs. NOSES-N0) = 0.549, HR(CLR-N0/NOSES-N0) = 1.271, Log-rank p (CLR-N1/N2 vs. NOSES-N1/N2) = 0.371, HR(CLR-N1/N2/NOSES-N1/N2) = 1.765. (**H**) disease-free survival after PSM: Log-rank p (CLR-N0 vs. NOSES-N0) = 0.378, HR(CLR-N0/NOSES-N0) = 1.408, Log-rank p (CLR-N1/N2 vs. NOSES-N1/N2) = 0.384, HR(CLR-N1/N2/NOSES-N1/N2) = 1.738.

From the follow-up data, there was no significant difference in OS and DFS between the two groups (Fig. 2A–D). Also, there were no significant differences in survival, recurrence and distant metastasis rates between the two groups at the follow-up endpoint (Table 5). We analyzed the long-term follow-up data of patients with different N stages in the two groups and found that there were no significant differences in DFS and OS between patients with the same N stage in the two groups (Fig. 2E–H).

**Table 5:** Comparison of the long-term outcomes of the two groups.

Outcome	Before PSM			After PSM		
	CLR (N = 106)	NOSES (N = 59)	*p* value	CLR (N = 59)	NOSES (N = 59)	*p* value
Status at the last follow-up (N%)			0.32			0.29
Survival	77(72.6%)	47(79.7%)		42(71.2%)	47(79.7%)	
Dead	29(27.4%)	12(20.3%)		17(28.8%)	12(20.3%)	
Local recurrence (N%)	10(9.4%)	4(6.8%)	0.56	5(8.5%)	4(6.8%)	0.73
Distant metastasis (N%)	11(10.4%)	7(11.9%)	0.77	7(11.9%)	7(11.9%)	1.00
Liver metastasis	9(8.5%)	5(8.5%)	/	6(10.2%)	5(8.5%)	/
Lung metastasis	2(1.9%)	2(3.4%)	/	1(1.7%)	2(3.4%)	/

## Discussion

Minimally invasive surgery is the direction of surgery. With the gradual development of minimally invasive surgery and functional surgery, it is more and more important to preserve the normal function of tissues and organs, reduce the impact of surgery on patients’ quality of life, and improve the quality of life of patients^8–12^. As the first center to carry out NOSES in China, we have been conducting it for about 7 years, so we have an advantage in case and follow-up data. Combining years of clinical experience, we retrospectively analyzed the NOSES of patients with low rectal cancer and the routine laparoscopy of transabdominal specimens in our department to illustrate and demonstrate the feasibility and safety of NOES in patients with low rectal cancer.

In this study, we used the PSM method to match baseline data for gender, age, BMI, ASA score, tumor size, preoperative CEA level, and T and N stage in both groups to achieve baseline data. Then we can similarly compare the perioperative data and postoperative follow-up information of the two groups of patients after PSM.

From the results of this study, for patients with low rectal cancer in the NOSES group, we stripped and removed the specimens in the presence of a sterile protective sleeve during the operation, and disinfected the anastomosis in time. According to postoperative patient data There was no significant difference in the incidence of postoperative abdominal infection between the NOSES group and the CLR group. It also confirmed that there was no significant difference in the asepsis of NOSES between the CLR group and the CLR group. Costantino et al. believed that the probability of NOSES may cause intra-abdominal infection was greater than conventional laparoscopy^13^. In our study, we found that there was no significant difference in postoperative infection between the NOSES group and the CLR group. Moreover, during the operation of the procedure, the sterile protective sleeve was placed before the specimen was disconnected, and a large amount of distilled water and physiological saline were washed before the end of the operation. Of course, there are many studies showed that NOSES is not a significant difference from conventional laparoscopy in terms of aseptic technique. It is a safe and feasible technique, which also supports our findings^14,15^.

Regarding the principle of tumor-free, we first observed that the detection of lymph nodes and the positive rate of double margins were not significantly different from the postoperative pathology of the two groups of patients. From the perspective of OS and DFS after surgery, there was no significant difference between the two groups. These also confirm that NOSES is consistent with the principle of tumor-free.

In addition, anal function at 6 months postoperatively and quality of life at 3 months postoperatively in patients with low rectal cancer are also important factors in assessing short-term outcomes after surgery. Costantino et al. suspected that NOSES may affect postoperative anal function in patients because the surgeon may destroy the anatomic structure of the anal sphincter during specimen extraction^13^. To discuss this issue, we decided to use the Wexner Incontinence Score to measure the patient's anal sphincter function. In this study, we found no significant difference in postoperative anal function between the two groups. For NOSES, the size of the tumor and the width of the specimen are limited in the indications, which better regulates and protects the postoperative anal function of the patient. The QLQ-C30 scale has good credibility and feasibility and can be used as a tool for assessing the quality of life of patients with malignant cancer. In this study, the NOSES group was superior to the CLR group in terms of global health status, physical function, role function and emotional function from the postoperative functional scale. From the patient's postoperative symptom scale, the NOSES group was significantly less than the CLR group in the pain, insomnia, constipation and diarrhoea.

We can see that the safety of NOSES seems to be no problem. Compared with the laparoscopic surgery with conventional transabdominal incision, the first and most intuitive manifestation is that there is no incision in the patient's abdomen, and the patient's satisfaction with the abdominal wall is better. Because there is no auxiliary incision, the consequent complications associated with the incision will not occur in patients of the NOSES group. For example, incisional hernia, fat liquefaction, incision redness, delayed healing, etc. can be avoided. From the results, we can also see that the degree of postoperative pain in patients with NOSES is greatly reduced, and the application of postoperative painkillers is also reduced. In the case that NOSES has so many benefits, it does not increase the hospitalization expenses of the patient.

The author acknowledges that there are certain limitations in this study. First of all, we admit that the technical errors caused by the different treatment groups are unavoidable. The skill of the operating surgeon is critical to the success of the procedure. This reason alone was impetus to credential our participating surgeons. So we can be sure that the doctors who perform the surgery are highly skilled and highly qualified. This study is a retrospective study and there must be some selectivity bias in patient enrollment. We used PSM to match the basic information of the enrolled patients, minimized their bias between the two groups. We can find that for patients with low rectal cancer, NOSES seems to have better short-term efficacy, and its long-term efficacy seems no differ with the application of conventional laparoscopic patients. Compared to other existing studies, our conclusions are the same. However, we have a more complete postoperative follow-up and survival analysis, and a larger sample size. We look forward to more prospective studies to study and consider the difference between NOSES and conventional laparoscopy surgery.

## Methods

### Selection of enrolled patients

All the patients diagnosed as low rectal cancer and underwent radical resection between January 2013 and June 2015 in the Colorectal Surgery Department of the Second Affiliated Hospital of Harbin Medical University were included in this study. These cases were all consecutive cases. Patients underwent surgery with NOSE were assigned as NOSES group while patients who received conventional laparoscopic-assisted resection were assigned as CLR group. A multiple disciplinary team (MDT) consisted of an endoscopist, an anesthetist and two surgeons would explain the two procedures thoroughly to the patients and the patients would make the final decision according to the suggestions. All the surgeries were performed in the same operating center by one experienced team. Surgeons were credentialed in both laparoscopic rectal cancer resection. Every surgeon has skillfully performed at least 100 cases of laparoscopic rectal cancer resections. All patients are informed on admission that all of their treatment-related information will be retained and may be used for scientific research. However, the patient's private information will be kept strictly confidential and the collection of information will not have any impact on the optimal treatment the patient receives. Patients provide informed consent for this. This study was approved by the Ethics Committee of the Second Affiliated Hospital of Harbin Medical University. All procedures in this study conformed to the ethical standards of our institution and were in accordance with the Declaration of Helsinki.

The inclusion criteria were as follow: (1) Patients aged between 18 and 80 years old; (2) Histopathology confirmed as rectal cancer and the distance between the tumor and the anus verge is no more than 5 cm; (3) Preoperative imaging examination (CT and MRI) suggested that the tumor diameter ≤ 5.0 cm and T stage was within T3; (4) Patients with BMI < 35 kg/m^2^; (5) Obtain informed consent of patients.

The exclusion criteria were as follow: (1) Contraindication of laparoscopic surgery; (2) Because of acute intestinal obstruction, perforation or bleeding and emergency surgery cases; (3) Combined with other organs of primary malignant cancer; (4) Patients with prophylactic ostomy or those with Ostomy for other reasons; (5) Patients with multiple primary colorectal cancer; (6) Patients accepted preoperative radiotherapy or chemotherapy; (7) Patients with medical history of cancer. (8) Incomplete information or loss of follow-up data.

We selected patients enrolled in the NOSES group and those enrolled in the CLR group based on the above criteria. Ultimately, 106 patients were included in the CLR group and 59 patients were included in the NOSES group.

### Surgical operation

Gastrointestinal preparation was performed before surgery. We recommend that patients start a semi-liquid diet 3 days before surgery and a liquid diet 1 day before surgery to reduce the production of bowel contents. The patient was given compound polyethylene glycol electrolyte solution orally and enema with clear water 12 h before surgery, and enema with clear water again 3 h before operation to clean the colorectum. After anesthesia, the patient was placed in the lithotomy position and a pneumoperitoneum was established. Insert a 10 mm trocar from the laparoscope and insert two 12 mm working trocars in the lower left quadrant and the lower right quadrant. Two 5 mm trocars were inserted in the upper left quadrant and the upper right quadrant. First, the liver, gallbladder, stomach, spleen, omentum, colon, small intestine, rectum and pelvic cavity were sequentially examined. Then the location of the tumor is explored. The lower rectal cancer is generally in a lower position. Most of the cancer are located below the peritoneal retraction. The surgeon can use the right hand to perform a rectal examination to meet with the operation forceps in left hand to determine the location and size of the tumor. After the release and dissection of the inferior mesenteric vein, the rectum and sigmoid colon are freed and the intestine below the tumor is shrunk. Finally, the NOSES group and the CLR group used different methods to perform specimen removal and digestive tract reconstruction.

In the CLR group, the specimen was taken through an auxiliary incision below the navel. And then the end-to-end anastomosis of the sigmoid colon and rectum is performed. Finally, the abdominal cavity was irrigated with sterile distilled water and normal saline, and the incision and the trocar hole were closed.

**Figure 3:**
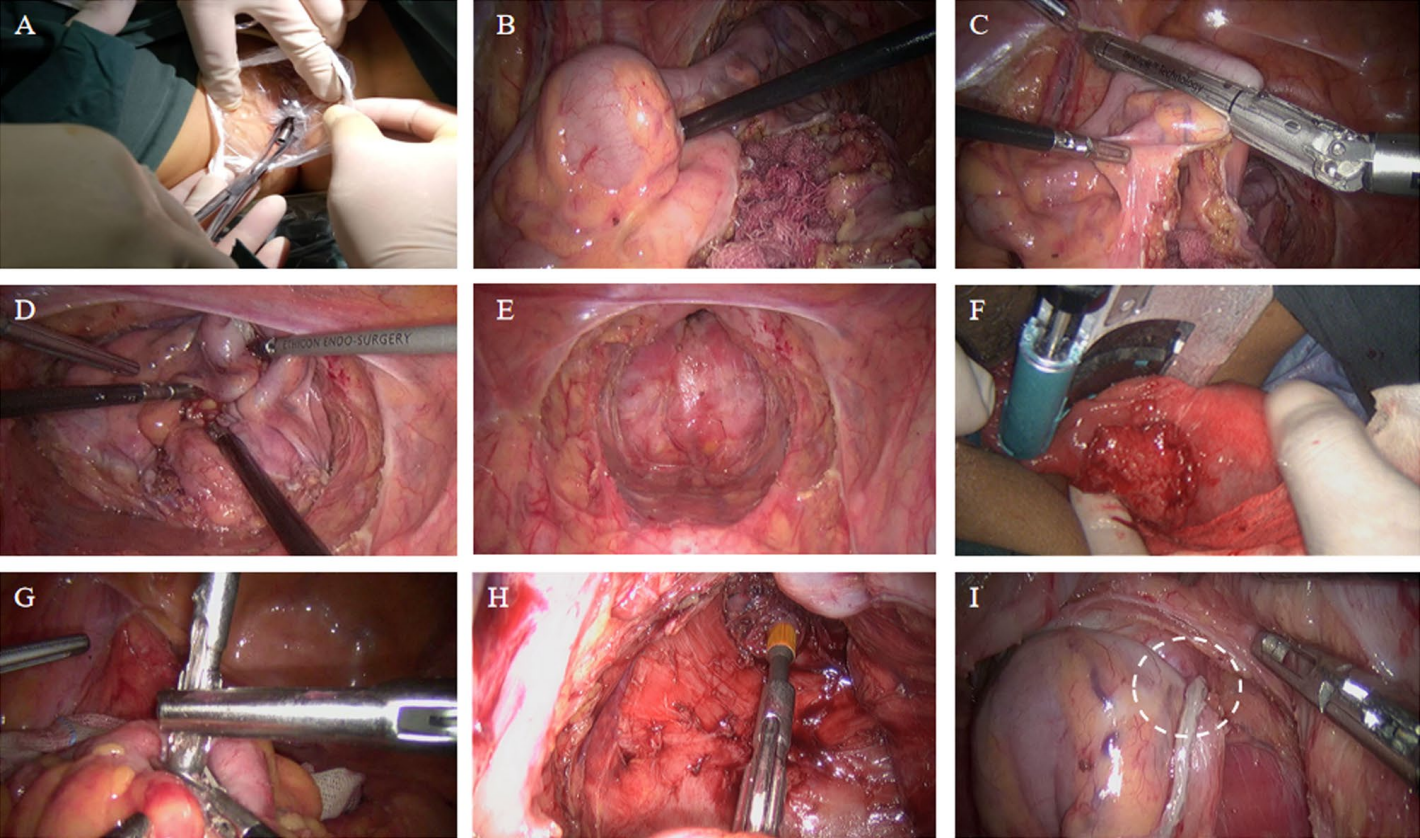
Disconnection and removal of specimens of the NOSES group. (**A**) Insert the sterile plastic protective sleeve through the anus to the top of the tumor 5 cm, and insert it into the anvil through the anus. (**B**) The anvil is delivered into the sigmoid colon. (**C**) The division of sigmoid colon is performed. (**D**) The specimen is extracted from anus. (**E**) Display of the pelvic after specimen extraction. (**F**) Use the Kaitu closure to cut the rectum at 1–2 cm below the tumor edge. (**G**) Remove the anvil connection rod at the broken end of the sigmoid colon. (**H**) Complete sigmoid rectal end-to-end anastomosis. (**I**) “Risk triangle”.

In the NOSES group, followed by gentle dilation of anal canal, the sterile plastic sleeve is inserted into the rectum, certifying that the upper edge of sleeve should be more than 5 cm above the upper edge of tumor. The anvil is then introduced into the bowel lumen through the sleeve, toward the proposed resection line of sigmoid colon (Fig. 3A,B). Proximal bowel division is performed through the right lower quadrant cannula using the 60 mm straight linear stapler (Fig. 3C), leaving the anvil inside of sigmoid colon. Povidone gauze is used to disinfect both sides of stumps. A large clamp is reintroduced into the bowel lumen through the anal canal to grab the rectal stump and to gently drag it out extracorporeally (Fig. 3D,E). The reverse rectal specimen is flushed extraabdominally with 1% povidone-iodine. The distal rectal resection is performed extraabdominally using the curved cutter stapler, making sure to preserve 1–2 cm of lower tumor margin, and the specimen is then removed (Fig. 3F). The rectal stump is then delivered back to pelvic cavity. Following the rectal dilation, the rectal irrigation is performed by using the 1% povidone-iodine. Then, the center rod of the anvil head is extracted from the proximal bowel lumen (Fig. 3G). The circular stapler is introduced transanally, and an end-to-end anastomosis is then performed with a great care, to certify the surrounding tissues not being caught in the anastomotic site (Fig. 3H,I). We recommend routine use of two drainage tubes near the anastomotic area in the pelvic cavity. For rectal cancer patients who undergo the ultra-low anastomosis surgery, the anastomosis should be firmly sutured extraabdominally. Finally, the abdominal cavity was irrigated with sterile distilled water and normal saline, and the trocar hole was closed.

### Data collection and follow-up

#### Data collection

In this study, all basic and treatment-related information of the patients was obtained from the medical record system of the Second Affiliated Hospital of Harbin Medical University. Patients were considered to have incision-related complications if their incision showed bleeding, fever, infection and fat liquefaction between 1 day and 2 weeks postoperatively, or if they developed an incisional hernia within 3 months postoperatively. In this study, patients were asked to fill out a Body Image Questionnaire (BIQ, see Supplementary Material 1) at 1 month after surgery, which we used to assess attitudes toward their physical appearance and satisfaction with the appearance of the scar^16^. We also applied the PFDI-20 to assess the extent to which patients' symptoms of lower urinary tract, lower gastrointestinal tract, and pelvic organ prolapse affected their quality of life before and at 3 months after surgery^17^. In order to investigate whether anal function was impaired in postoperative patients, we used the Wexner Incontinence Score^18^ (Supplementary Table 1) to assess patients’ anal function at 6 months postoperatively. EORTC QLQ-C30 is the core scale of the Quality of Life Measurement Scale System for Cancer Patients developed by the European Organization for Research and Treatment (EORTC) to determine the quality of life of all cancer patients^19^. In the present study, we used this score to assess the quality of life of patients 3 months after surgery.

#### Follow-up

Patients who underwent surgery in our department were followed up every 3 months for two years after surgery and every 6 months for the following three years until the patient died or the study was terminated. If the patient does not return to the observation, the information is obtained by letter or telephone. All patients were followed up to death or the end of the study in June 2020. Therefore, all patients were followed for at least 60 months or until death.

### Statistical analysis

PSM was used to balance the baseline data between two groups because of its ability to reduce selection bias. Propensity score matching was performed on a 1:1 ratio based on baseline information including gender, age, BMI, ASA score, preoperative CEA, T stage, N stage, preoperative PFDI-20 scores. A logistic regression model was used to analyze the variable assignment of the baseline data in 118 patients and the caliper value was 0.2. The quantitative data were expressed as the mean ± SD and compared using Student’s t-test. For continuous data such as postoperative VAS scores, we used a two-factor repeated measures ANOVA for comparison. Categorical variables were expressed using the Chi-square test or Fisher’s exact probability method. *p* < 0.05 was considered statistically significant.

## Supplementary Information

Supplementary Information.
