# Supplementary material for: Comparison of natural orifice specimen extraction surgery and conventional laparoscopic-assisted resection in the treatment effects of low rectal cancer
Source: Sci Rep. 2021 Apr 29;11:9338. doi: 10.1038/s41598-021-88790-8 (PMC8085046; doi:10.1038/s41598-021-88790-8)
Supplement: Supplementary file 1 — Supplementary Information. [file 41598_2021_88790_MOESM1_ESM.doc]

**Comparison of Natural orifice specimen extraction surgery and conventional laparoscopic-assisted resection in the treatment effects of low rectal cancer**

Yihao Zhu*^1^• Huan Xiong*^1^ • Yinggang Chen, MD^1^• Zheng Liu, MD^2^• Zheng Jiang, MD^2^• Rui Huang, MD^1^• Feng Gao, MD^1^• Qian Zhang, MD^1,3^• Meng Wang, MD^3^• Yinghu Jin, MD^1^• Tianyu Qiao^1^• Tianyi Ma^1^• Hanqing Hu, MD^1^• Xishan Wang, MD^1,2^• Qingchao Tang*, MD^1^• Guiyu Wang*, MD^1,3^

1 Department of Colorectal Surgery, the Second Affiliated Hospital of Harbin Medical University, Harbin 150081, China

2 Department of Colorectal Surgery, National Cancer Center, National Clinical Research Center for Cancer, Cancer Hospital, Chinese Academy of Medical Sciences and Peking Union Medical College, Beijing 100021, China

3 Department of Colorectal Surgery, Department of Colorectal Surgery, Zhejiang Cancer Hospital (Affiliated Cancer Hospital of the Chinese Academy of Sciences), Hangzhou 310022, China

**Correspondence:**

Qingchao Tang, MD, Department of Colorectal Surgery, the Second Affiliated Hospital of Harbin Medical University, Harbin 150081, China. Tel: +86-13945052628. Email: 13945052628@qq.com.

Guiyu Wang, MD, Department of Colorectal Surgery, Zhejiang Cancer Hospital (Affiliated Cancer Hospital of the Chinese Academy of Sciences), Hangzhou 310022, China. Tel: +86 15545153577. Email: guiywang@163.com.

These authors contributed equally: Yihao Zhu, Huan Xiong

**Supplementary material 1:**

**The body image questionnaire:**

1. Are you less satisfied with your body since the operation?
   range: 1=no, not at all
    2= a little bit
    3= quite a bit
    4= yes, extremely
   2. Do you think the operation has damaged your body?
   range: 1=no, not at all
    2= a little bit
    3= quite a bit
    4= yes, extremely

3. Do you feel less attractive as a result of your disease or treatment?
range: 1=no, not at all
 2= a little bit
 3= quite a bit
 4= yes, extremely

4. Do you feel less feminine/masculine as a result of your disease or treatment?
range: 1=no, not at all
 2= a little bit
 3= quite a bit
 4= yes, extremely

5. Is it difficult to look at yourself naked?
range: 1=no, not at all
 2= a little bit
 3= quite a bit
 4= yes, extremely

6. On a scale from 1 to 7, how satisfied are you with your (incisional) scar?

| 1= very unsatisfied | 2 | 3 | 4= not unsatisfied/ not satisfied | 5 | 6 | 7= very satisfied |
| --- | --- | --- | --- | --- | --- | --- |

7. On a scale from 1 to 7, how would you describe your (incisional) scar?

| 1= revolting | 2 | 3 | 4= not revolting/ not beautiful | 5 | 6 | 7= beautiful |
| --- | --- | --- | --- | --- | --- | --- |

8. Could you score your own incisional scar on a scale from 1 to 10?

Supplementary table 1: Wexner Incontinence Score

| Type of Incontinence | Frequency | | | | |
| --- | --- | --- | --- | --- | --- |
|  | NEVER | Rarely | Some-  times | Usually | Always |
| Solid | 0 | 1 | 2 | 3 | 4 |
| Liquid | 0 | 1 | 2 | 3 | 4 |
| Gas | 0 | 1 | 2 | 3 | 4 |
| Wears pad | 0 | 1 | 2 | 3 | 4 |
| Lifestyle alteration | 0 | 1 | 2 | 3 | 4 |

0 = perfect. 20 = complete incontinence.

Never = 0 (never). Rarely ≤ l/month. Sometimes ≤ l/week, ≥ l/month. Usually ≤ l/day, ≥ l/week. Always ≥ l/day.
